# Caregiver use of MUAC tapes in South Sudan: a three-group prospective comparison

**DOI:** 10.3389/fnut.2024.1324063

**Published:** 2024-02-06

**Authors:** Shannon Doocy, Sule Ismail, Emily Lyles, Chiara Altare, Sarah Bauler, Francis Obali, Daniel Atem, Eva Leidman, The South Sudan MUAC Study Team

**Affiliations:** ^1^Johns Hopkins Bloomberg School of Public Health, Baltimore, MD, United States; ^2^US Centers for Disease Control and Prevention, Juba, South Sudan; ^3^World Vision International, London, United Kingdom; ^4^World Vision South Sudan, Juba, South Sudan; ^5^US Centers for Disease Control and Prevention, Atlanta, GA, United States

**Keywords:** mid-upper arm circumference, community management of acute malnutrition, screening by mothers, acute malnutrition, South Sudan

## Abstract

**Introduction:**

Nutrition program modifications occurred globally in response to the COVID-19 pandemic. Within community management of acute malnutrition (CMAM), community screenings for acute malnutrition were replaced by caregivers monitoring child mid-upper arm circumference (MUAC), but questions remain about different MUAC tapes’ performance and acceptability for caregiver use.

**Methods:**

The study was conducted in Central Equatoria and Warrap States, South Sudan, between March 2022 and January 2023. A three-group prospective non-randomized design was used to compare the performance of three MUAC tapes (UNICEF 2009, UNICEF 2020, and GOAL MAMI) used by caregivers. The primary outcome was the false negative rate (i.e., the proportion of children not identified as wasted by the caregiver but classified as wasted by enumerators). Caregivers with children aged 5–53 months were assigned to and trained on the use of 1 of the 3 tapes and followed for 8 months, including three monitoring visits and baseline/endline surveys.

**Results:**

Of the 2,893 enrolled children, 2,401 (83.0%) completed baseline, endline, and two or more monitoring visits. Only 3.7% of children were identified as wasted by caregivers and 3.8% by study team measurement. Cumulative measurement agreement between caregivers and enumerators was similar by tape. False negative and false positive rates were both <0.5% overall and similar among the tapes. There were differences in training needs and durability between the tapes, but all three were acceptable and performed equally well.

**Discussion:**

Caregiver measurement of child MUAC is feasible in South Sudan. The three MUAC tapes were acceptable, and caregivers could measure accurately with minimal support. All tapes performed similarly and are appropriate for use in Family MUAC programs in South Sudan. There were indications that the UNICEF 2020 tape may be less durable; the GOAL MAMI tape has the added benefit of being suitable for assessments of infants <6 months of age.

## 1 Introduction

The COVID-19 pandemic has impacted population health tremendously both directly and via secondary impacts such as reductions in service availability and willingness to seek care. While COVID-19 morbidity and mortality impacts were limited in children, indirect effects, including disruptions to health services such as vaccinations, nutrition, and antenatal care, translated to significant increases in child deaths (1, 2). With some of the poorest child health indicators globally, ongoing conflict, weak infrastructure, widespread floods, and declining food security, South Sudan is a country where the secondary impacts of COVID-19 are of great concern. At the onset of the pandemic, almost half of the country’s population was expected to face a crisis or, worse, acute food insecurity (3, 4). The food security situation further deteriorated in 2020 due to COVID-19-related movement restrictions and quarantines, rising staple food prices, a weak economy, and the continued impacts of conflicts and floods (3, 4). Estimates indicated that more than 313,000 children would experience severe acute malnutrition [SAM; defined as <−3 Z-scores of the median WHO weight-for-height growth standards (WHZ), MUAC <11.5 cm, or the presence of bilateral pitting edema], and more than 1 million children would experience moderate acute malnutrition (MAM; defined as ≥ − 3 and < −2 WHZ or MUAC ≥11.5 cm and < 12.5 cm) in 2021 (5).

Community management of acute malnutrition (CMAM) programs are designed to identify children with acute malnutrition and to help them recover. These programs are implemented on a widespread basis globally and within the context of South Sudan and are typically operated by non-governmental organizations (NGOs). To reduce contact and COVID-19 transmission, CMAM programs were adopted globally, impacting screening, identification, and treatment of acutely malnourished children (6). The South Sudan Nutrition Cluster issued adapted guidelines for CMAM programs in the context of COVID-19 at the end of March 2020, which included various changes to service delivery intended to reduce contact and risk (7). The modifications included suspending nutrition screening in favor of the Family MUAC approach, where caregivers are issued a MUAC tape and trained to take MUAC measurements on their children and then self-refer to a community health worker or nutrition program site.

The Family MUAC approach has been used in several settings before the pandemic, and while the approach is generally considered acceptable, feasible, and reliable, evidence is limited, and concerns about measurement error and low sensitivity persist (8–11). These have prompted the testing of modified designs that are intended to make tapes easier to use (9, 11). Evidence suggests that modified MUAC tapes increase the accuracy and precision of measurement compared to the Standard UNICEF MUAC tape and that wider tapes and alternative closures may be associated with increased sensitivity (12, 13). The 2020 UNICEF tape, which, to the best of our knowledge, has not been previously evaluated, and the MAMI tape (by GOAL) are new designs that have been specifically developed for use by caregivers within Family MUAC programs (14, 15). In particular, the 2020 UNICEF tape has user instructions on the reverse side to guide caregivers. The MAMI tape takes a more simplified approach without millimeter measurements and a color-scheme-only designation that may be more appropriate for illiterate caregivers.

This study evaluated the performance, acceptability, and feasibility of three different MUAC tapes within the context of ongoing Family MUAC programs to provide actionable recommendations for improving nutrition programs in South Sudan.

## 2 Materials and methods

**Figure 1:**
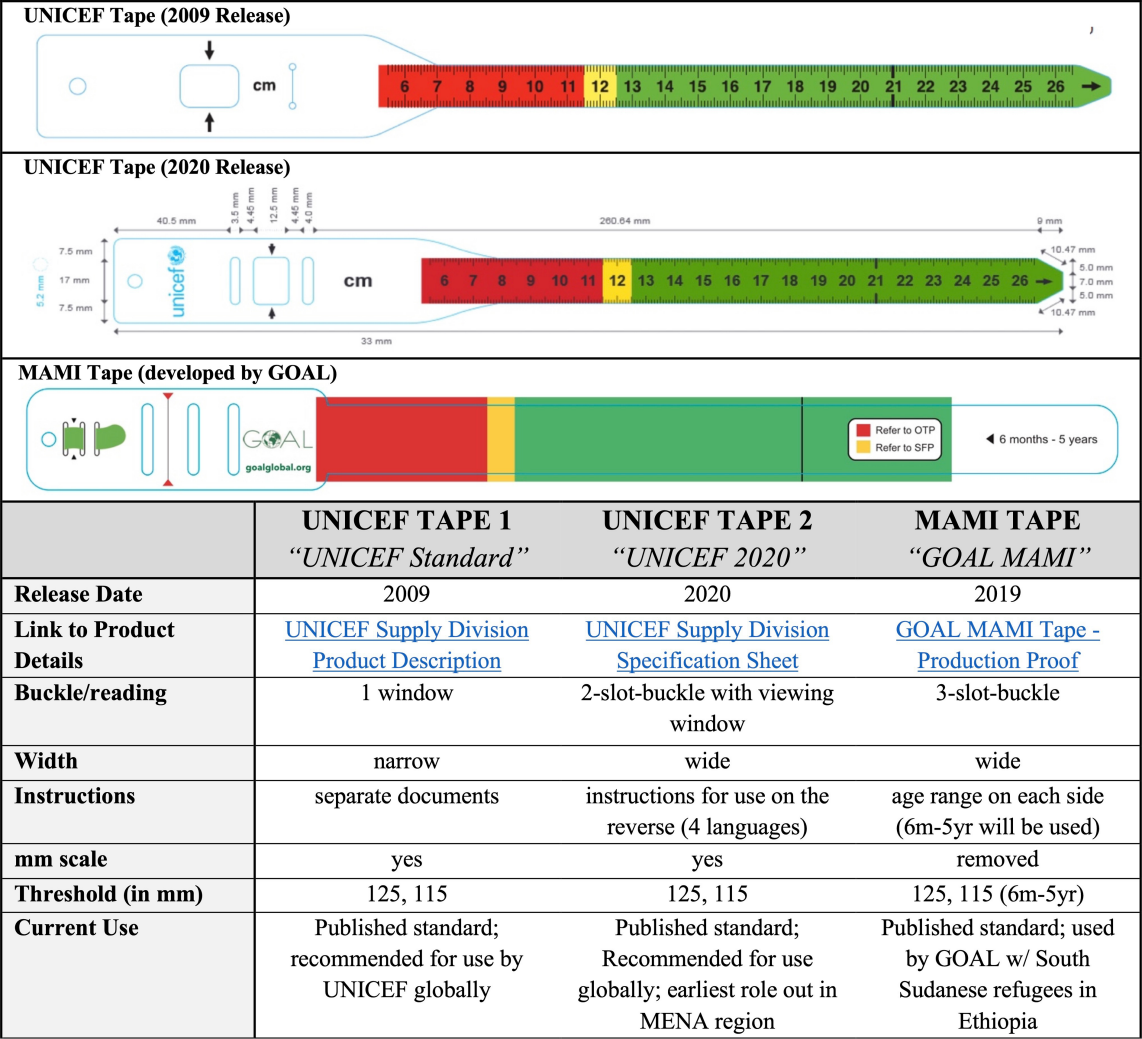
MUAC measurement devices included in the evaluation. Note that cutoffs for diagnosing acute malnutrition are the same on all tapes, but images are slightly different scales.

**Figure 2:**
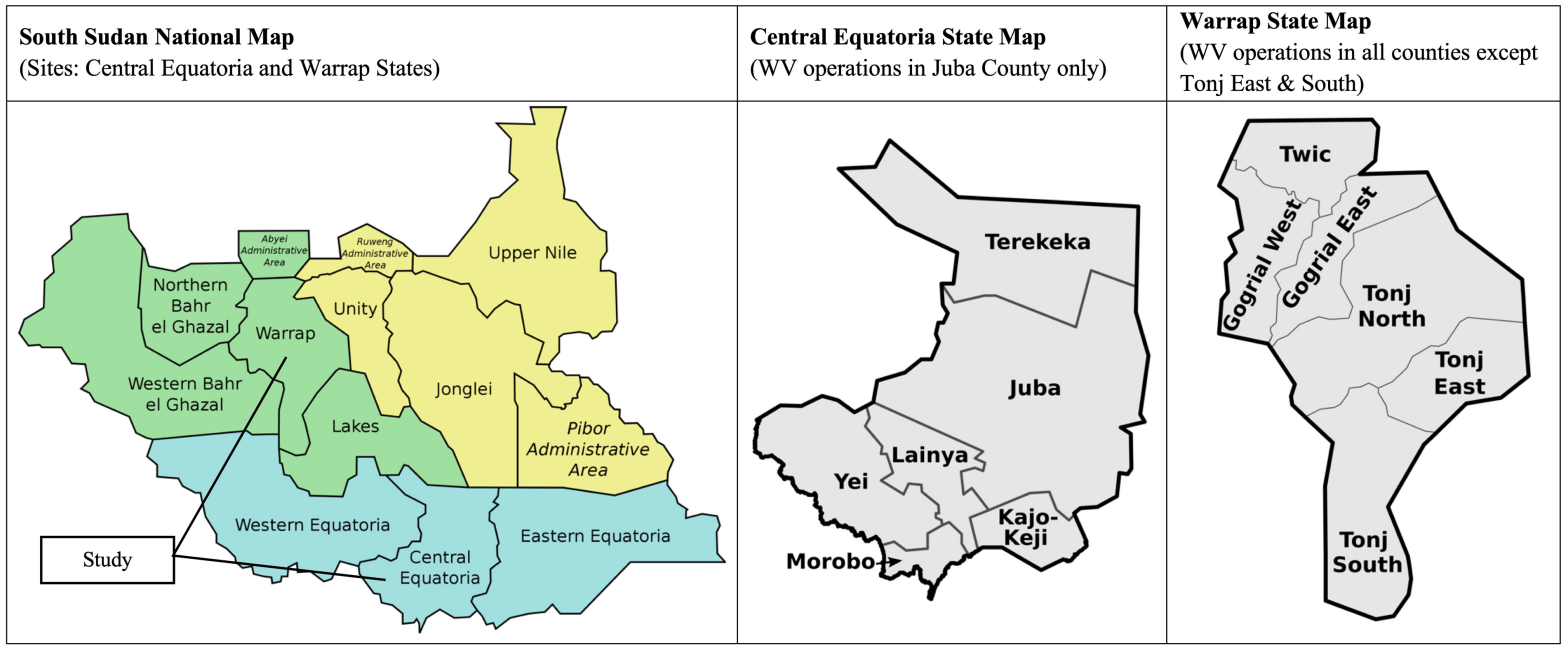
Map of study locations.

A three-group prospective non-randomized design was used to compare the performance of the three MUAC tapes (Figure 1). The tapes are similar in function but Figure 2 have different design styles. The main differences include width, the window for reading the result, the number of slot buckles, and the presence of numbers and instructions. The primary outcome was the false negative rate (i.e., the proportion of children not identified as acutely malnourished by the caregiver were defined as acutely malnourished by study enumerators). Frequency of measurement and perceptions of tape use were secondary objectives captured both quantitatively and qualitatively to ascertain participant perspectives on the acceptability of the different devices.

The study was conducted in Central Equatoria (Juba County) and Warrap (Twic County) states in catchment areas of seven CMAM program sites operated by World Vision (Figure 2). Sites rather than individuals were assigned a MUAC tape to avoid sharing the tapes by neighboring households. Community selection was based on the following criteria: presence of an ongoing Family MUAC program, adequate caseload, and anticipated continuity of access (e.g., no ongoing conflict or recurrent flooding). Each CMAM program site was assigned 1 of the tapes, and all households with children 5–53 months of age within the selected communities within the catchment area were eligible to participate. Caregivers were invited to attend a 1–2 h training by World Vision nutrition staff, where they were provided with the respective tape and trained on its use. At the end of the session, program staff confirmed caregivers could take accurate measurements. Those who consented to participate in the study completed an enrollment interview and were followed for 8 months. Study contacts included three monitoring visits that were each approximately 8 weeks apart and an endline survey, with no additional formal training of caregivers during the follow-up period. Within each household, the same individual/caregiver performed MUAC measurements for the duration of the study. Tapes were not replaced during the study period; however, at the end of the study, UNICEF 2020 and Goal MAMI tapes were replaced with UNICEF 2009 tapes because these are the only tapes approved for use in Family MUAC programming in South Sudan.

### 2.1 Sample size

Calculations were based on the following assumptions: (1) power (1-β) of 80% and significance of *α* = 0.05; (2) 2-sided comparison of the 2020 UNICEF tape or the MAMI tape vs. Standard UNICEF tape to assess device performance; (3) baseline false negative rates ranging from 10 to 30% (based on program data); (4) a minimum detectable difference of 10% in the false negative rate (determined to be programmatically relevant); (5) design effect of 1.5 to take into account differences across CMAM program sites; and (6) 20–30% loss to follow-up over the study period. A minimum required sample size of 2,100 households (700 per group) was identified as sufficient to detect differences ≥10% in the false negative rate.

### 2.2 Enrollment and data collection

Inclusion criteria included residence within selected communities in the CMAM catchment area, presence of a 5- to 53-month-old child in the household, and consent to participate in the study. The 5–53 months age range was selected because CMAM programs target children 6–59 months of age. The study was originally planned for 6 months, allowing all children to remain within this age range for the full study period. Due to low acute malnutrition rates, the study length was extended to 8 months (one additional monitoring visit). Children who were acutely malnourished at enrollment were excluded from the study and referred to the CMAM program for treatment. Quantitative data were collected 5 times over the 8-month study period: at baseline, in 3 monitoring visits (months 2, 4, and 6), and at endline (month 8). The study period went from March to December 2022 in Central Equatoria and from April 22 to January 23 in Warrap. The baseline survey questionnaire included individual (mother and child) and household level characteristics (e.g., size and displacement status), including household head sex and educational attainment; living conditions and routine expenditures; receipt of food, cash transfers, and other humanitarian assistance; household food security; use of the measurement tapes; and recent illnesses and care-seeking for child(ren) aged 6–59 months. Instruments for monitoring visits and the endline interview were more limited and focused on food security indicators, tape utilization, and child anthropometry. At each data collection time point, caregivers were first observed taking a measurement, and then a trained enumerator followed to take the “gold standard” measurement. As part of training, enumerators underwent a standardization exercise to ensure accuracy in MUAC measurements.

Focus group discussion (FGD) participants were caregivers who participated in the quantitative evaluation, and two types of FGDs were designed: (1) “single tape groups” focused on barriers and facilitators of tape use, with participants belonging to the same study arm and sharing experiences about the tape they were given, and (2) “comparative tape groups,” with caregivers who were given different tapes that aimed to compare experiences across tapes. A total of 20 FGDs were conducted, including 12 single tape groups (4 per tape, 2 per site) and 8 comparative tape groups (4 per site). FGDs were conducted in month 7 of the study; each FGD had 6 participants and was conducted in Juba Arabic. FGDs were recorded with participants’ agreement, transcribed, and translated into English by the note-taker and facilitator.

### 2.3 Data analysis

Statistical analysis was conducted in Stata 15 (College Station, TX). Descriptive statistics were used to examine baseline characteristics as well as tape use and caregiver/study team measurement agreement over the study period for each measurement tape group. The statistical significance of differences by tape type was assessed using chi-square and *t*-test methods. Analyses included all children who completed the study (defined as having completed baseline, endline, and 2 or more monitoring visits) and their respective households. A total of 133 children were excluded from the analysis for the following reasons: the child was found to be wasted upon enrollment, age at enrollment fell outside study eligibility parameters or was above 61 months at endline, and the child no longer qualified for nutrition treatment. Statistically significant differences in many household characteristics and some outcomes were also seen between the two states, which was not unanticipated given that they represent the comparison of very rural areas of South Sudan to greater Juba. Differences by state that are not illustrated in the tables in the main manuscript are provided in Supplementary Tables S1, S2.

Children were classified as wasted if their MUAC measurement was in the yellow or red category (MUAC < 12.5 cm) at monitoring 1, 2, 3, and/or endline; the green category (MUAC > 12.5 cm) was not wasted. Comparison of caregiver measurement to the study team gold standard was based on color category. Measurement agreement was evaluated as true negative [children correctly identified as not wasted (specificity)], true positive [children correctly identified as wasted (sensitivity)], false negative (children incorrectly identified as not wasted), or false positive (children incorrectly identified as wasted).

For the qualitative component, acceptability was defined as the caregiver’s satisfaction with MUAC tape attributes, and feasibility was defined as the caregiver’s ease in using the MUAC tapes correctly. Using a deductive and inductive approach, descriptive, process, emotional, value, and concept codes were developed to capture perceptions about the acceptability and feasibility of the tapes. Once the transcripts were coded, coded segment reports were extracted, and a saturation grid was developed to map and count the occurrence of emerging themes and sub-themes. Qualitative data were managed and coded in MAXQDA (Berlin, Germany).

### 2.4 Ethical approvals

The study was reviewed and approved by the South Sudan Ministry of Health Ethics Committee and Institutional Review Board at Johns Hopkins Bloomberg School of Public Health. This activity was reviewed by the US Centers for Disease Control and Prevention (CDC) and was conducted consistent with applicable federal law and CDC policy.

## 3 Results

A total of 2,893 children from 2,185 households were enrolled in the study, including 975 (33.7%) from households who received GOAL MAMI tapes, 953 (32.9%) who received UNICEF 2020 tapes, and 965 (33.4%) who received standard UNICEF tapes. Of the enrolled children, 2,401 (83.0%) from 1,826 households completed baseline, endline, and 2 or more monitoring visits and, as such, were included in analyses [800 (33.3%) received GOAL MAMI tapes, 775 (32.3%) received UNICEF 2020 tapes, and 826 (34.4%) received standard UNICEF tapes]. Study completion rates were significantly higher in Warrap than in Central Equatoria, where the population is more mobile (*p* < 0.001); however, there was no significant difference in the proportion of available children who completed the study (i.e., did not permanently move away or die) across the three comparison groups (*p* = 0.613).

Baseline characteristics of participating children were similar across groups and states with respect to gender and age. The age at enrollment was 28.9 months, and 51.4% of children were female (Table 1). MUAC at enrollment was 14.6 cm on average overall and, though significantly higher for children in the UNICEF 2020 group, group mean MUAC at enrollment only ranged from 14.5 to 14.7 cm. Mean MUAC at enrollment was also significantly higher in Central Equatoria (14.9 cm) than in Warrap (14.4 cm). Significantly more children in the UNICEF Standard group (24.5%) had previously been diagnosed with acute malnutrition than those in the other groups (16.5% GOAL MAMI and 16.3% UNICEF 2020). Prior acute malnutrition diagnoses were also more common in Warrap (21.3%) than in Central Equatoria (16.0%).

**Table 1:** Participant characteristics at baseline by state and study group.

		Overall (*N* = 2,401)	By location			By tape type			
			Central Equatoria (*n* = 981)	Warrap (*n* = 1,420)	*p*-value	GOAL MAMI (*n* = 800)	UNICEF 2020 (*n* = 775)	UNICEF standard (*n* = 826)	*p*-value
Participant (child) characteristics									
Female		1,233 (51.4%)	522 (53.2%)	711 (50.1%)	0.130	415 (51.9%)	396 (51.1%)	422 (51.1%)	0.937
Age (in months)	Mean (SD)	28.9 (13.5)	28.9 (13.4)	29.0 (13.5)	0.772	29.6 (13.3)	28.3 (13.6)	29.0 (13.4)	0.186
6 to <24 months		846 (35.2%)	358 (36.5%)	488 (34.4%)	0.283	259 (32.4%)	284 (36.6%)	303 (36.7%)	0.116
≥ 24 months		1,555 (64.8%)	623 (63.5%)	932 (65.6%)		541 (67.6%)	491 (63.4%)	523 (63.3%)	1,555 (64.8%)
MUAC at enrollment (cm)^1^	Mean (SD)	14.6 (1.1)	14.9 (1.1)	14.4 (1.0)	***<0.001***	14.6 (1.1)	14.7 (1.1)	14.5 (1.1)	**0.001**
Prior acute malnutrition diagnosis		460 (19.2%)	157 (16.0%)	303 (21.3%)	**0.001**	132 (16.5%)	126 (16.3%)	202 (24.5%)	***<0.001***
Household characteristics		*(N = 1,826)*	*(n = 750)*	*(n = 1,076)*		*(n = 608)*	*(n = 607)*	*(n = 611)*	
Household size	Mean (SD)	7.5 (2.5)	7.4 (2.8)	7.6 (2.2)	0.073	7.4 (2.4)	7.5 (2.3)	7.8 (2.6)	**0.005**
Household is currently displaced		144 (7.9%)	137 (18.3%)	7 (0.7%)	***<0.001***	49 (8.1%)	50 (8.2%)	45 (7.4%)	0.365
Female-headed household		547 (30.0%)	205 (27.3%)	342 (31.8%)	**0.041**	169 (27.8%)	170 (28.0%)	208 (34.0%)	**0.026**
Received food assistance (past month)^2^		79 (4.3%)	7 (0.9%)	72 (6.7%)	***<0.001***	30 (4.9%)	27 (4.4%)	22 (3.6%)	0.511
Household Hunger Scale	Mean (SD)	2.8 (1.4)	2.5 (1.2)	3.1 (1.4)	***<0.001***	3.0 (1.3)	2.8 (1.3)	2.7 (1.5)	**0.012**
Little to no hunger		248 (13.9%)	136 (18.9%)	112 (10.6%)	***<0.001***	59 (10.0%)	73 (12.4%)	116 (19.2%)	***<0.001***
Moderate hunger		1,240 (69.6%)	546 (75.7%)	694 (65.5%)		414 (70.2%)	441 (75.0%)	385 (63.8%)	
Severe hunger		293 (16.5%)	39 (5.4%)	254 (24.0%)		117 (19.8%)	74 (12.6%)	102 (16.9%)	
Caregiver characteristics									
Caregiver sex									
Male		42 (2.3%)	25 (3.3%)	17 (1.6%)	**0.014**	9 (1.5%)	7 (1.2%)	26 (4.3%)	***<0.001***
Female		1784 (97.7%)	725 (96.7%)	1,059 (98.4%)		599 (98.5%)	600 (98.8%)	585 (95.7%)	
Caregiver age (years)^3^	Mean (SD)	28.0 (7.8)	29.1 (7.8)	27.0 (7.7)	***<0.001***	27.6 (7.2)	28.1 (7.8)	28.1 (8.4)	0.532
≤ 18 years		204 (11.3%)	76 (10.5%)	128 (11.9%)	***<0.001***	67 (11.2%)	76 (12.7%)	61 (10.1%)	0.075
19–31 years		331 (18.4%)	136 (18.7%)	195 (18.1%)		108 (18.0%)	116 (19.4%)	107 (17.7%)	
32–37 years		1,125 (62.4%)	484 (66.6%)	641 (59.6%)		386 (64.3%)	370 (61.8%)	369 (61.2%)	
38–48 years		43 (2.4%)	15 (2.1%)	28 (2.6%)		11 (1.8%)	10 (1.7%)	22 (3.6%)	
49+ years		99 (5.5%)	16 (2.2%)	83 (7.7%)		28 (4.7%)	27 (4.5%)	44 (7.3%)	
Highest level of education completed									
Less than primary		1,440 (82.0%)	549 (73.2%)	891 (88.6%)	***<0.001***	491 (84.7%)	484 (83.9%)	465 (77.6%)	**0.013**
Primary		181 (10.3%)	94 (12.5%)	87 (8.6%)		48 (8.3%)	53 (9.2%)	80 (13.4%)	
Secondary or higher		135 (7.7%)	107 (14.3%)	28 (2.8%)		41 (7.1%)	40 (6.9%)	54 (9.0%)	

^1^Per study team measurement. ^2^Includes in-kind food assistance and cash assistance that could be used to purchase food. ^3^Specific age (in years) only reported by 1,584 (86.7%) respondents and age categories were developed according to local memorable events to facilitate more reliable recall. Bold values in indicate statistical significance at *p*<0.05.

Household size was larger among those receiving the UNICEF Standard tape but similar by state. More households in Central Equatoria (18.3%) were currently displaced compared to those in Warrap (0.7%) (Table 1). Female-headed households were more common in Warrap (31.8%) than Central Equatoria (27.3%) and among those receiving the UNICEF Standard tape (34.0% vs. 28.0% UNICEF 2020 and 27.8% GOAL MAMI). Only 4.3% of households received food or cash assistance in the month before enrollment; while similar by tape type, most households that received food assistance were in Warrap. Regarding household food security, severe hunger was significantly higher in Warrap (24.0%) than in Central Equatoria and in the GOAL MAMI group (19.8% vs. 16.9% of UNICEF Standard and 12.6% of UNICEF 2020). Caregivers were similar in all tape groups in terms of age, but significantly more caregivers in the GOAL MAMI (84.7%) and UNICEF 2020 (83.9%) groups completed less than primary education than in the UNICEF Standard group (77.6%). Caregivers were, on average, older and completed more schooling in Central Equatoria than in Warrap.

### 3.1 Tape use

Caregivers’ self-reported comfort using the measuring tapes was similar by tape type, with more than 97% of caregivers reporting confidence at each time period (Table 2). The study team’s perception of caregiver confidence using tapes was more varied; however, with only 81.5% of caregivers in the GOAL MAMI group perceived to be comfortable at monitoring visit 1 (compared to 98.4% for UNICEF 2020 and 97.0% for UNICEF Standard, *p* < 0.001) (Table 2). At monitoring visit 2, only 87.7% of caregivers in the UNICEF 2020 group were perceived to be comfortable using the tape (vs. 98.0% for GOAL MAMI and 98.3% for UNICEF Standard, *p* < 0.001). These proportions increased to 97% or higher at monitoring visit 3 and endline. Differences across groups were significant at the endline, with all caregivers in the GOAL MAMI group believed to be comfortable using the tapes (*p* < 0.001).

**Table 2:** Caregiver use of MUAC tapes during monitoring visits and endline.

				Caregiver is comfortable using tape		Caregiver measurement frequency				Caregivers measuring less than monthly
			N (%)^1^	Self-reported by caregiver	Study staff perception	At least Weekly	At least monthly	At least once since last visit	Not since last visit	
Monitoring 1	Overall		1729 (94.7%)	98.4%	90.6%	90.7%	8.2%	0.8%	0.4%	19 (1.0%)
	By tape type	GOAL MAMI	575 (94.6%)	98.3%	81.5%	88.8%	9.2%	1.3%	0.7%	11 (1.8%)
		UNICEF 2020	581 (95.7%)	99.0%	98.4%	92.7%	6.8%	0.2%	0.4%	3 (0.5%)
		UNICEF standard	573 (93.8%)	97.9%	97.0%	90.5%	8.5%	1.0%	0.0%	5 (0.8%)
		*p*-value		0.296	***<0.001***	0.095				0.063
Monitoring 2	Overall		1802 (98.7%)	99.7%	94.7%	91.7%	6.3%	1.5%	0.5%	33 (1.8%)
	By tape type	GOAL MAMI	598 (98.4%)	99.7%	98.0%	93.7%	4.3%	1.4%	0.5%	11 (1.8%)
		UNICEF 2020	600 (98.8%)	99.5%	87.7%	86.5%	11.0%	1.7%	0.8%	13 (2.1%)
		UNICEF standard	604 (98.9%)	100.0%	98.3%	94.6%	3.7%	1.5%	0.2%	9 (1.5%)
		*p*-value		0.244	***<0.001***	***<0.001***				0.681
Monitoring 3	Overall		1770 (97.0%)	99.9%	97.6%	92.2%	5.6%	2.0%	0.2%	34 (1.9%)
	By tape type	GOAL MAMI	592 (97.4%)	100.0%	97.6%	94.7%	3.8%	1.5%	0.0%	8 (1.3%)
		UNICEF 2020	587 (96.9%)	99.8%	97.9%	89.9%	7.9%	2.0%	0.2%	11 (1.8%)
		UNICEF standard	591 (96.7%)	99.8%	97.4%	91.9%	5.3%	2.5%	0.4%	15 (2.5%)
		*p*-value		0.602	0.872	0.062				0.337
Endline	Overall		1825 (100%)	99.8%	98.6%	91.8%	7.4%	0.5%	0.3%	13 (0.7%)
	By tape type	GOAL MAMI	608 (100%)	100.0%	100.0%	94.2%	5.6%	0.2%	0.0%	1 (0.2%)
		UNICEF 2020	606 (100%)	99.8%	98.7%	89.4%	10.0%	0.6%	0.0%	3 (0.5%)
		UNICEF standard	611 (100%)	99.7%	97.0%	91.5%	6.8%	0.8%	0.9%	9 (1.5%)
		*p*-value		0.370	***<0.001***	**0.004**				**0.018**

^1^Percent of households with available children measured at time point (excludes households in which all children died or permanently moved away at or before time point). Bold values in indicate statistical significance at *p*<0.05.

**Figure 3:**
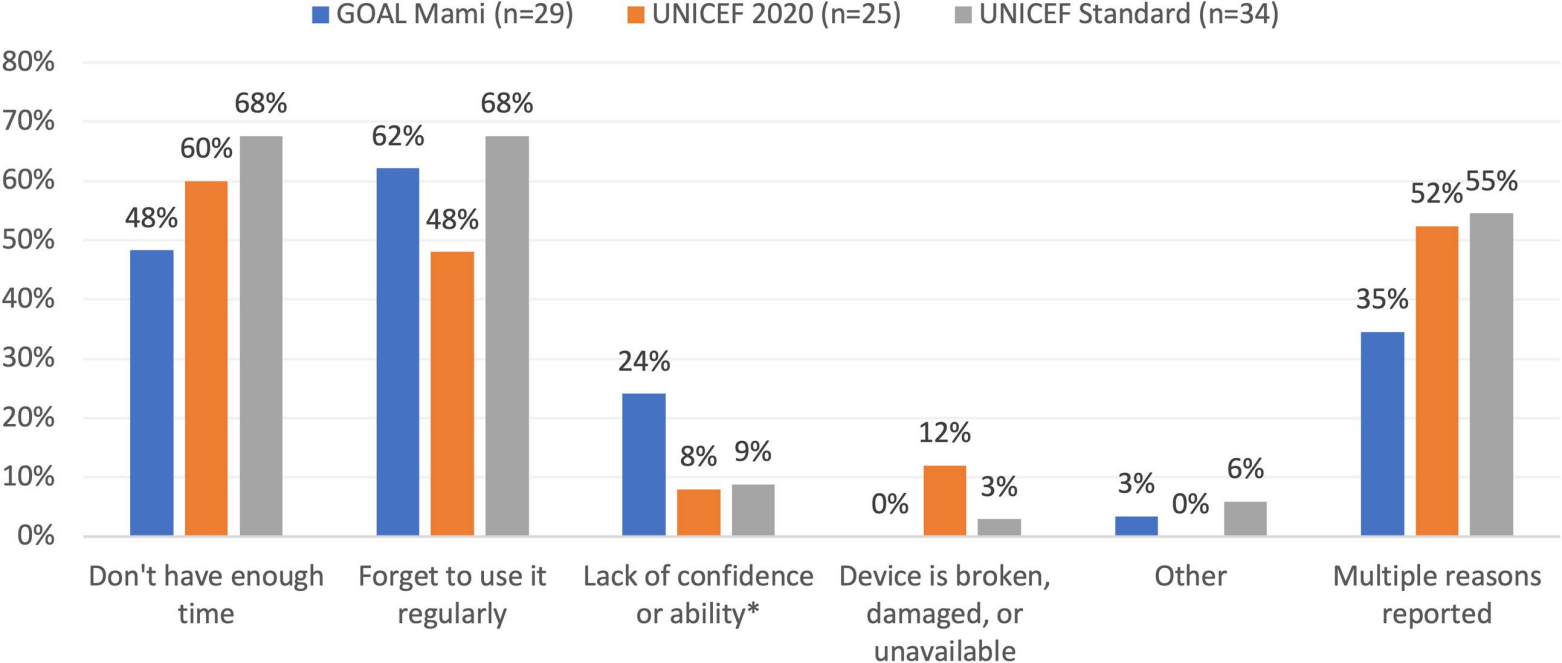
Reasons for infrequent measurement among households measuring less than monthly. *Includes device is hard to use, worried about measuring incorrectly, or not receiving enough training.

Caregivers in all groups reported that they frequently measured children using the tapes; more than 86% in each group reported weekly measurements at each time point (Table 2). Fewer than 2% of caregivers at each time point reported measuring their child less than monthly, and this was similar across groups at all monitoring visits. At the endline, 9 (1.5%) households that received the UNICEF Standard tape measured their children less than monthly compared to 3 (0.5%) households receiving UNICEF Standard and 1 (0.2%) household that received the GOAL MAMI tape (*p* = 0.018). The most frequently reported reasons for measuring children less than monthly were that caregivers did not have enough time or forgot to use it regularly. Smaller numbers of caregivers also reported infrequent measurement because they lacked confidence or ability or because their device was broken, damaged, or unavailable (Figure 3).

### 3.2 Children’s MUAC measurement

Children’s MUAC measurements during monitoring visits and endline, including caregiver and study team measures and measurement agreement, are presented in Table 3. Mean MUAC as measured by caregivers was similar between the two UNICEF tape groups (the GOAL MAMI tape does not measure exact MUAC). The color classification was also similar across groups at the first two monitoring visits and endline (range: 98.0–99.4%). At monitoring visit 3, however, significantly fewer children in the UNICEF 2020 group (97.7%) were classified in the green category compared to those in the other groups (99.5% GOAL MAMI, 99.1% UNICEF Standard; *p* = 0.002). Mean MUAC, as measured by the study team, was 14.5 cm overall at monitoring visits 1 and 3 and at endline, but nominally higher (14.7 cm) at monitoring visit 2. At the last two monitoring visits and endline, children in the UNICEF Standard group had significantly higher mean MUAC as measured by the study team (range: 14.6–14.8 cm) compared to those in both the UNICEF 2020 (range: 14.5–14.6 cm) and GOAL MAMI (range: 14.3–14.5 cm) groups (*p* < 0.001 for all three time points). More than 97% of children in all groups were classified by the study team as falling in the green category throughout the study period. This differed by tape type only at monitoring visit 3 when 98.1% of children in the UNICEF 2020 group were classified as green compared to 98.9% for UNICEF Standard and 99.4% for GOAL MAMI (*p* = 0.047).

**Table 3:** Child MUAC measurement during monitoring visits and endline (caregiver, study team, and measurement agreement).

				Caregiver measurement				Evaluation team measurement				Measurement agreement^2^			
			N (%)^1^	Mean MUAC (SD)	Green	Yellow	Red	Mean MUAC (SD)	Green	Yellow	Red	True negative	True positive	False negative	False positive
Monitoring 1	Overall		2,254 (93.9%)	14.6 (1.3)	98.0%	1.8%	0.2%	14.5 (1.1)	97.8%	2.0%	0.2%	97.6%	1.9%	0.4%	0.2%
	By tape type	GOAL MAMI	741 (92.6%)	–	97.8%	1.8%	0.4%	14.5 (1.1)	97.4%	2.0%	0.5%	97.3%	2.0%	0.5%	0.1%
		UNICEF 2020	741 (95.6%)	14.6 (1.2)	98.3%	1.6%	0.1%	14.6 (1.1)	98.0%	2.0%	0.0%	97.8%	1.6%	0.4%	0.1%
		UNICEF Standard	772 (93.5%)	14.6 (1.3)	97.8%	2.1%	0.1%	14.5 (1.1)	97.9%	2.1%	0.0%	97.6%	1.9%	0.1%	0.3%
		*p*-value		0.992	0.742			0.871	0.102			0.855			
Monitoring 2	Overall		2,358 (98.2%)	14.9 (1.2)	98.5%	1.4%	0.0%	14.7 (1.1)	98.4%	1.4%	0.2%	98.3%	1.3%	0.3%	0.2%
	By tape type	GOAL MAMI	788 (98.5%)	–	98.6%	1.4%	0.0%	14.5 (1.0)	98.6%	1.1%	0.3%	98.6%	1.4%	0.0%	0.0%
		UNICEF 2020	761 (98.2%)	14.9 (1.2)	98.2%	1.7%	0.1%	14.6 (1.1)	97.9%	1.8%	0.3%	97.6%	1.6%	0.5%	0.3%
		UNICEF Standard	809 (97.9%)	15.0 (1.3)	98.8%	1.2%	0.0%	14.8 (1.1)	98.8%	1.2%	0.0%	98.5%	1.0%	0.2%	0.2%
		*p*-value		0.702	0.601			***<0.001***	0.446			0.288			
Monitoring 3	Overall		2,325 (96.9%)	15.0 (1.1)	98.8%	1.1%	0.1%	14.5 (1.0)	98.8%	1.1%	0.1%	98.6%	1.0%	0.2%	0.2%
	By tape type	GOAL MAMI	778 (97.2%)	–	99.5%	0.5%	0.0%	14.3 (1.0)	99.4%	0.6%	0.0%	99.4%	0.5%	0.1%	0.0%
		UNICEF 2020	750 (96.9%)	15.0 (1.1)	97.7%	2.3%	0.0%	14.5 (1.0)	98.1%	1.9%	0.0%	97.6%	1.7%	0.1%	0.5%
		UNICEF Standard	797 (96.5%)	15.0 (1.1)	99.1%	0.6%	0.3%	14.6 (1.0)	98.9%	0.9%	0.3%	98.9%	0.9%	0.3%	0.0%
		*p*-value		0.666	**0.002**			***<0.001***	**0.047**			**0.022**			
Endline	Overall		2,400 (100%)	14.8 (1.1)	99.4%	0.5%	0.1%	14.5 (1.0)	99.2%	0.7%	0.1%	99.2%	0.6%	0.2%	0.0%
	By tape type	GOAL MAMI	800 (100%)	–	99.4%	0.5%	0.1%	14.4 (1.1)	98.9%	1.0%	0.1%	98.9%	0.6%	0.5%	0.0%
		UNICEF 2020	774 (100%)	14.8 (1.2)	99.2%	0.5%	0.3%	14.6 (1.0)	99.1%	0.8%	0.1%	99.1%	0.8%	0.1%	0.0%
		UNICEF Standard	826 (100%)	14.8 (1.1)	99.6%	0.4%	0.0%	14.6 (1.0)	99.6%	0.4%	0.0%	99.6%	0.4%	0.0%	0.0%
		*p*-value		0.906	0.664			***<0.001***	0.482			0.168			

^1^Percent of available children measured at time point (excludes children who died or permanently moved away at or before time point). ^2^ Comparison of caregiver measurement to study team gold standard based on category, where green (>12.5 cm) is not wasted and yellow (11.5–12.5 cm)/red (<11.5 cm) is wasted; True Negative = children correctly identified as not wasted (specificity), True Positive = children correctly identified as wasted (sensitivity), False Negative = children incorrectly identified as not wasted, False Positive = children incorrectly identified as wasted. Bold values in indicate statistical significance at *p*<0.05.

Given low wasting rates, most children were classified as true negatives when comparing caregiver measurement to that performed by the study team. Specificity was highest at the endline, increasing overall during each follow-up time point from 97.6% at monitoring visit 1 to 99.2% at the endline. False negatives were uncommon, accounting for only 0.2–0.4% of children at each visit. False positives accounted for only 0.2% of children at all monitoring visits and no children at endline. Measurement agreement was similar by tape type at all time points except monitoring visit 3, when children in the UNICEF 2020 group saw significantly fewer true negatives (97.6%) and more positives, both true (1.7%) and false (0.5%) compared to the other groups (*p* = 0.022).

### 3.3 Cumulative identification of wasting

Key outcomes of interest measured cumulatively over the study period are presented in Table 4. Of the 2,401 children completing the study, 3.7% overall were diagnosed to be wasted at least once during the study period [i.e., classified in the yellow or red category (MUAC < 12.5 cm) at monitoring 1, 2, 3, and/or endline] based on caregiver measurements and 3.8% were ever wasted based on study team measurements. The proportions of children ever wasted per caregiver and study team measurement were nominally higher for children in households receiving the UNICEF 2020 tape, but these did not significantly differ from the other groups (caregiver measure *p* = 0.566; study team measure *p* = 0.662). The proportion of children ever wasted was, however, significantly higher in Central Equatoria than in Warrap, both per caregiver (4.7 and 3.0%, respectively; *p* = 0.026) and study team (5.4 and 2.7%, respectively; *p* = 0.001) measurements. Cumulative measurement agreement between caregivers and the study team was also similar by tape type (*p* = 0.734), but specificity was significantly higher and sensitivity was lower in Warrap (96.9 and 2.6%, respectively) compared to Central Equatoria (94.1 and 4.2%, respectively) (*p* = 0.001).

**Table 4:** Cumulative identification of wasting over the study period.

	Overall	By location			By tape type			
		Central equatoria	Warrap	*p*-value	GOAL MAMI	UNICEF 2020	UNICEF standard	*p*-value
Total completing the study	2,401	981	1,420		800	775	826	
% of children enrolled	83.0%	67.5%	98.6%	***<0.001***	82.1%	81.3%	85.6%	**0.028**
% of available children^1^	96.2%	91.7%	99.6%	***<0.001***	96.5%	96.5%	95.7%	0.613
Ever wasted^2^								
Caregiver measurement	3.7%	4.7%	3.0%	**0.026**	3.4%	4.3%	3.4%	0.566
Study team measurement	3.8%	5.4%	2.7%	**0.001**	3.9%	4.3%	3.4%	0.662
Measurement agreement^3^								
True negative (specificity)	95.8%	94.1%	96.9%	**0.001**	96.0%	95.1%	96.1%	0.734
True positive (sensitivity)	3.2%	4.2%	2.6%		3.2%	3.6%	2.9%	
False negative	0.6%	1.2%	0.1%		0.6%	0.6%	0.5%	
False positive	0.4%	0.5%	0.4%		0.1%	0.6%	0.5%	
Measurement tape status								
Ever damaged	20.6%	16.2%	23.8%	***<0.001***	11.0%	11.2%	4.9%	***<0.001***
Always available and functional	69.5%	50.0%	83.6%	***<0.001***	75.9%	62.9%	69.8%	***<0.001***

^1^Excludes children who died or permanently moved away. ^2^Classified in the yellow or red category (MUAC<12.5 cm) at monitoring 1, 2, 3, and/or endline. ^3^Comparison of caregiver measurement to study team gold standard based on category, where green is not wasted and yellow/red is wasted; true negative = children correctly identified as not wasted (specificity); true positive = children correctly identified as wasted (sensitivity); false negative = children incorrectly identified as not wasted; false positive = children incorrectly identified as wasted. Bold values in indicate statistical significance at *p*<0.05.

### 3.4 Tape availability and durability

**Figure 4:**
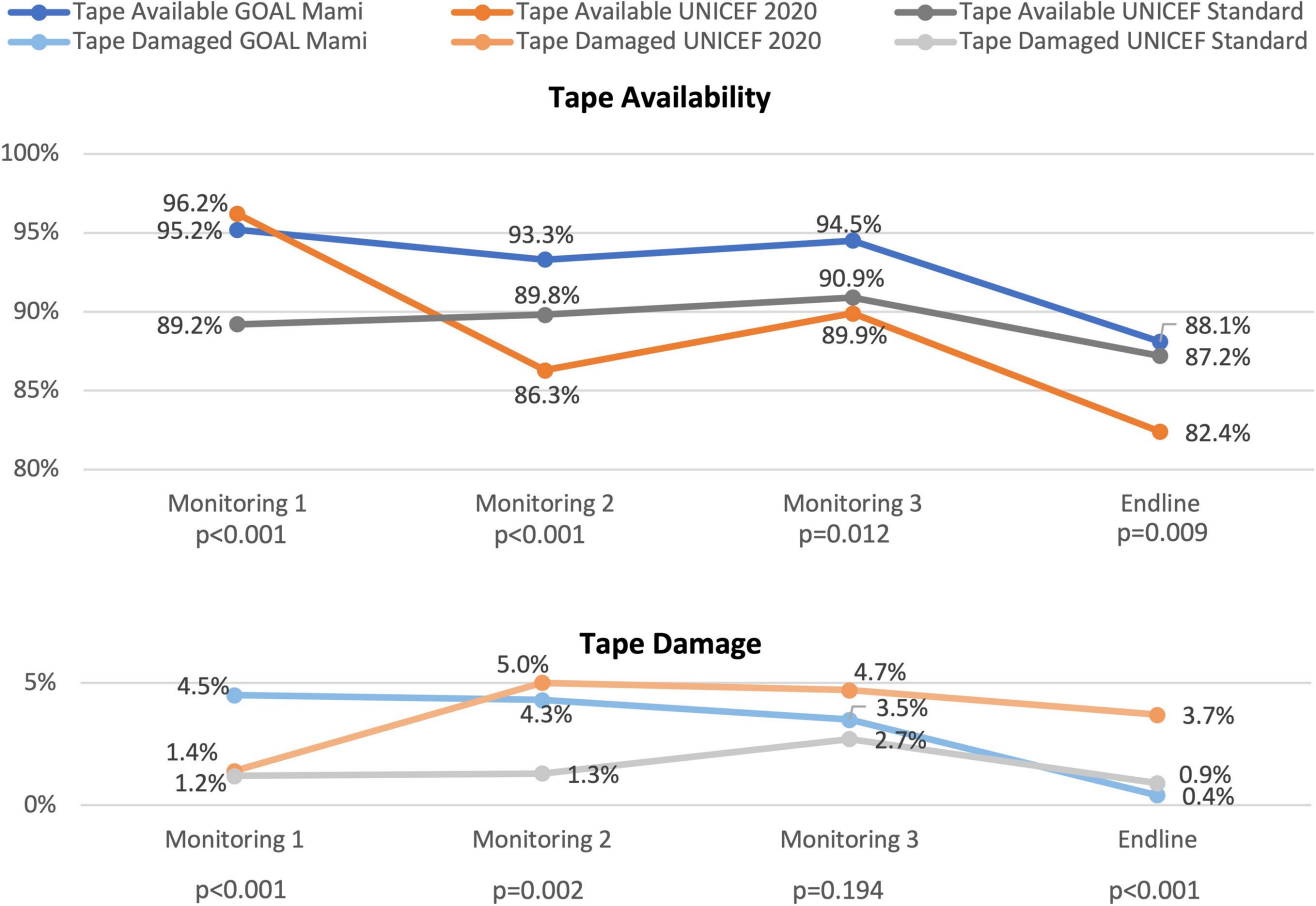
Tape status over the study period. *P*-values indicate group differences in availability/damage at indicated time point.

**Figure 5:**
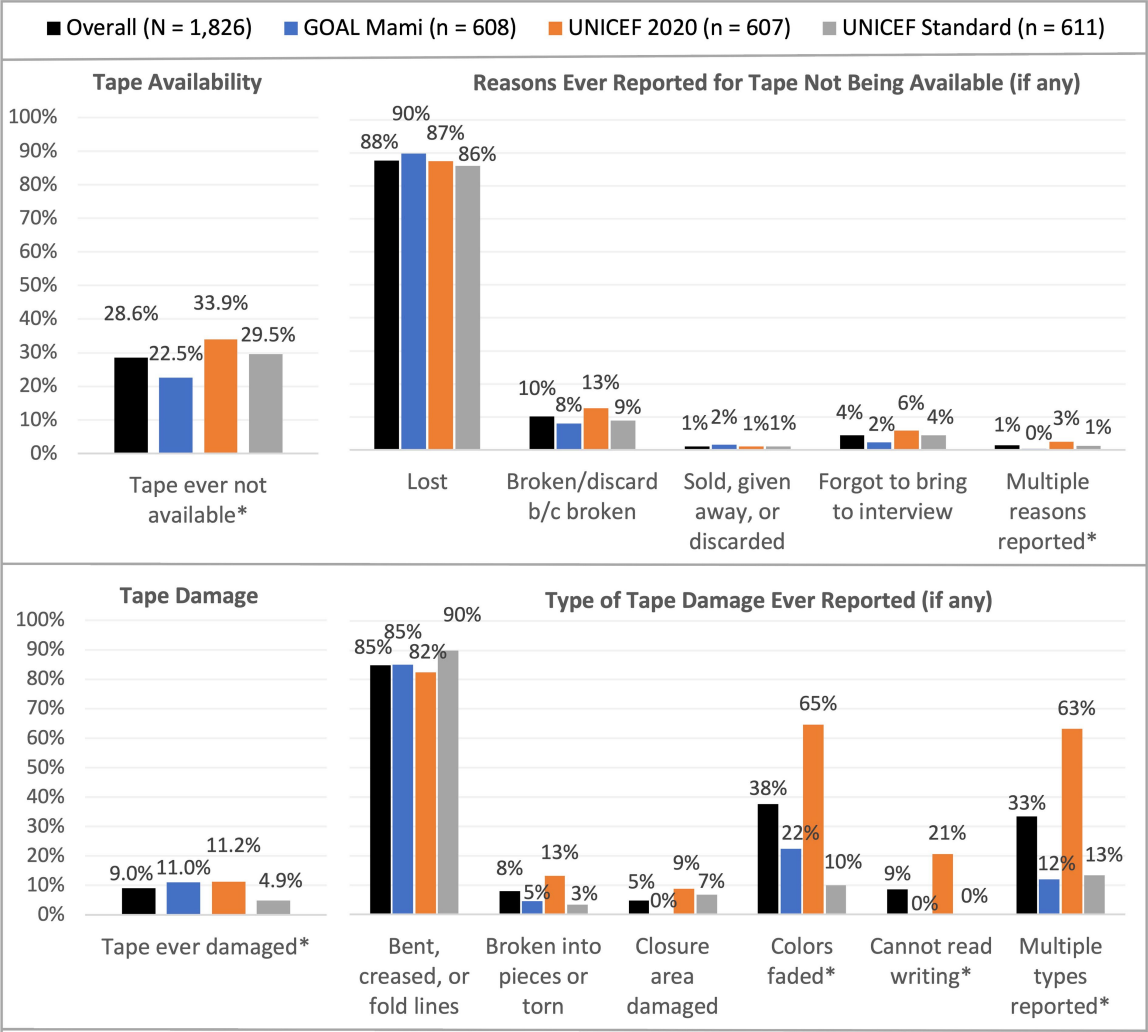
Tape availability and damage. Reasons for unavailability and damage type categories are not mutually exclusive (multiple selection permitted). Lost tapes were mostly temporarily misplaced and then located again. *Statistically significant differences across groups (*p* < 0.05).

Measurement tapes were often, though not always, available and functional during study team follow-up visits. Cumulatively, only 69.5% of households overall always had available and functional measurement tapes (Table 4), and tape availability at each time point ranged from 86.2 to 93.5% and was lowest at the endline. This proportion was higher in Warrap (83.6% vs. 50.0% in Central Equatoria, *p* < 0.001) and in the GOAL MAMI group (75.9% vs. 69.8% in UNICEF Standard and 62.9% in UNICEF 2020, *p* < 0.001). Tape availability at each monitoring and endline visit is summarized in Figure 4 and cumulative tape availability is presented in Figure 5. Tapes were reported as [ever] not available in significantly more households in the UNICEF 2020 tape group (33.9%) than in the UNICEF Standard (29.5%) and GOAL MAMI (22.5%) groups (*p* < 0.001). By far, the most common reason for tapes not being available, reported by 87.6% of households, was that they were lost.

Tape damage was relatively common, with 20.6% of households ever reporting damage to their tape during the study period, but fewer than 5.0% reporting damage at each data collection time point (Table 4). Tape damage at each monitoring and endline visit is summarized in Figure 4 and cumulatively in Figure 5. Damage was reported by more households in Warrap (23.8%) than Central Equatoria (16.2%) (*p* < 0.001) and by fewer households in the UNICEF Standard group (4.9%) compared to the UNICEF 2020 (11.2%) and GOAL MAMI (11.0%) groups (*p* < 0.001). The most frequently reported type of damage during the study was that the tape was bent, creased, or had permanent fold lines (reported by 84.8% of the 165 total households with any damage). Fewer tapes had faded colors (37.6% overall), though fading was significantly more common for the UNICEF 2020 tape (64.7% with damaged tapes) compared to GOAL MAMI (22.4%) and UNICEF Standard (10.0%) tapes (*p* < 0.001). Households also reported the UNICEF 2020 tapes being broken into pieces/torn (13.2%) more than the other tapes (4.5% GOAL MAMI, 3.3% UNICEF Standard), and the UNICEF 2020 was the only tape for which households reported being unable to read the writing (20.6%). More households reported multiple types of damage over the study period for the UNICEF 2020 tape (63.2% with damaged tapes) compared to the others (11.9% GOAL MAMI, 13.3% UNICEF Standard; *p* < 0.001).

### 3.5 Qualitative results

In general, the acceptability of the tapes was high, with caregivers perceiving the tapes as flexible and thick but sturdy enough not to tear up. Participants described keeping the tapes as clean and safe as possible, and a main challenge was keeping them away from children who may consider them as toys (especially the GOAL MAMI tape, which was bigger). Caregivers kept the tapes under the pillow or mattresses, hung them up high, or stored them in handbags, books, or drawers. Shorter tapes were perceived as easier to keep safe. Participants felt that using the tape was easy, thanks to the training they received. Using the slits correctly was not easy for all caregivers, and training was required, especially for the MAMI tape that has three slits. Instructions on the new UNICEF tape were considered easy to understand and follow, as were colors used on all tapes (green, yellow, and red) to indicate the child’s nutritional status. One-fifth of respondents reported having difficulties with numbers and millimeters.

## 4 Discussion

This study compares caregiver use of three MUAC tape designs (i.e., 2009 UNICEF tape, 2020 UNICEF tape, and the GOAL MAMI tape) in South Sudan. The performance of the three MUAC tapes was similar and satisfactory across study sites, and caregivers were able to consistently measure arm circumference correctly. There was 99.2% enumerator-caregiver agreement, and at the endline, 90.1% of caregivers reported measuring child MUAC at least weekly. Our results provide evidence that with minimal training (less than 2 h), caregivers were equipped with the knowledge and skills to monitor child MUAC. Although caregivers observed enumerators taking MUAC measurements, they did not receive supportive supervision and refresher training from Community Nutrition Volunteers (CNVs), though this is a key component of the standard Family MUAC approach. Qualitative results found tapes to be highly acceptable and feasible to use among caregivers. These results are relevant for a range of locations with high acute malnutrition prevalence, including those inaccessible due to conflict or other reasons, locations with an absence of CNVs or a robust community health worker platform, or where routine nutrition screenings did not return following the COVID-19 pandemic.

The findings are consistent with several studies that provide evidence of the feasibility of caregiver screening for acute malnutrition. In Niger, a non-randomized evaluation found that mothers can screen their children for acute malnutrition accurately and frequently with minimal training (9). Another study in Niger found that engaging caregivers in at-home surveillance of children with uncomplicated SAM was highly feasible and that caregivers could accurately perform MUAC measurement after a short training by a nurse (16). A study in Kenya found that both Click-MUAC bracelets and modified MUAC tapes performed well when used by caregivers, with no significant differences in specificity between Click-MUAC bracelets and modified MUAC tapes and high sensitivity for detecting SAM (13). Finally, a recent review that focused on community platforms for the detection and treatment of SAM concluded that scaling up MUAC monitoring by caregivers and CHWs to detect SAM is a promising step toward improving coverage of SAM detection, diagnosis, and treatment (11).

MUAC tapes were generally durable and remained usable but with some expected wear and tear. Of note, the UNICEF 2020 tape was least likely to be available and most likely to be damaged at the endline. Of the three tapes, the UNICEF 2020 tape was most likely to be reported as broken into pieces or torn, having a damaged closure area, faded colors, and illegible writing. The UNICEF 2020 tape is described as disposable, eco-friendly, and made from biodegradable paper, and was developed in the early phases of the COVID-19 pandemic (17). The eco-friendly disposable design likely contributed to the lack of durability; however, considering tapes were designed for short-term use, they held up remarkably well over the course of the study period. While the logistics of more frequent tape replacement could reduce tape availability and coverage, decreasing costs of local production could offset the costs of more frequent tape replacement and the need for additional tapes. The use of more durable materials could also be considered, given that COVID-19 is unlikely to be transmitted on surfaces and is not a major concern for children.

The UNICEF 2020 MUAC tapes used in this study were produced by UNICEF and obtained directly from the UNICEF supply division. This is relevant because UNICEF encouraged local production of tapes at the time of their release, making it likely that there will be variations in tape performance due to differences in materials and production (18). Other researchers have noted discrepancies in measurement between different types of MUAC tapes and observed that measurement errors can occur due to differences in the thickness of the tape material. Systematic differences/bias in MUAC measurements taken using different tapes had implications for the identification of SAM cases, where some children were classified as not malnourished due to measurement error (~2 mm) when tape thickness was not taken into account during design. Study authors called for global standards for MUAC tape design and standardization of reporting, in particular, the fixed thickness of MUAC tapes, shifting the measurement ruler/colors on tapes of non-standard thickness to account for the difference, and calibrating tapes similar to standard practice for other anthropometric measurement devices (19).

## 5 Limitations

The central limitation of this study was lower than anticipated rates of acute malnutrition. Prevalence rates from prior surveys and estimates shared by the South Sudan nutrition cluster both suggested that global acute malnutrition (GAM, or the presence of both MAM and SAM) rates would be higher; however, GAM rates at baseline were 3.8 and 1.0% in Central Equatoria and Warrap, respectively, and incident cases of SAM and MAM remained low throughout the study period (20). Low GAM rates meant there were fewer opportunities for women to (in)correctly measure a wasted child, and the small number of wasted children in the sample reduced the study’s ability to detect measurement errors within this important sub-population. Another limitation was difficulty in following populations over time and attaining high coverage levels at monitoring visits. This was a particular challenge in Juba, where many children left their villages for schooling or care by relatives elsewhere, and in several locations in Twic County, where flooding hindered access. While not necessarily a limitation, it is important to note the differences in study populations and sites. Most importantly, Twic County is rural, had higher levels of food insecurity, and experienced flooding during the study, whereas Juba County is urban, had greater health service availability, and a more mobile population.

## 6 Conclusion

Caregivers in South Sudan were able to consistently and accurately use MUAC tapes to monitor children aged 6–59 months for acute malnutrition over an 8-month period. The three MUAC tapes that were compared all performed well and were acceptable to caregivers. The UNICEF 2020 MUAC tape was slightly less durable than the other tapes, and it performed remarkably well given its intended disposable design. In the face of shortages of CNVs and health workers, caregiver provision of MUAC tapes along with a brief training may be a successful approach to increasing coverage of nutrition screening and acute malnutrition case finding. While tapes performed equally well and are suitable for use for the Family MUAC approach, the UNICEF 2020 tape may be the least advantageous due to lesser durability and the increased risk for measurement differences if locally produced. The UNICEF 2009 tape, which is currently used in South Sudan, and the GOAL MAMI tape may be more suitable. The added benefit of the GOAL MAMI tape is that it also can be used to monitor younger infants. In summary, caregiver monitoring of MUAC is feasible in South Sudan and should be considered in addition to other approaches to increase coverage of acute malnutrition screening.

## Data availability statement

The dataset supporting the conclusions of this study can be found in the Humanitarian Data Exchange at: https://data.humdata.org/dataset/ssd-family-muac-mainpaper.

## Ethics statement

The studies involving humans were approved by the South Sudan Ministry of Health Ethics Committee and the Institutional Review Board at Johns Hopkins Bloomberg School of Public Health. The studies were conducted in accordance with the local legislation and institutional requirements. The ethics committee/institutional review board waived the requirement of written informed consent for participation from the participants or the participants’ legal guardians/next of kin because an oral consent process in the local language was used because of high levels of illiteracy, as it would not be appropriate to have participants sign a consent document they cannot read.

## Author contributions

SD: Conceptualization, Funding acquisition, Investigation, Methodology, Project administration, Supervision, Writing – original draft, Writing – review & editing. SI: Investigation, Supervision, Writing – review & editing. ELy: Formal analysis, Writing – original draft, Writing – review & editing. CA: Formal analysis, Investigation, Supervision, Writing – original draft, Writing – review & editing. SB: Formal analysis, Investigation, Supervision, Writing – original draft, Writing – review & editing. FO: Investigation, Writing – review & editing. DA: Investigation, Supervision, Writing – review & editing. ELe: Conceptualization, Funding acquisition, Investigation, Software, Writing – review & editing. The South Sudan MUAC Study Team: Writing – review & editing.

## The South Sudan MUAC Study Team

The South Sudan MUAC Study Team includes World Vision South Sudan staff members Francis Obali, Daniel Atem, and Subek Peters who managed data collection, the study enumerators and Epiu Stephen Leonard who liaised between the study team and World Vision Nutrition Program Staff. Maya Ramaswamy and Sarah King from the US Centers for Disease Control and Prevention provided data quality monitoring and data cleaning support. Sandra Banks, Prachi Srivastava, and Julia Hussian from Johns Hopkins School of Public Health supported the qualitative analysis. Finally, Kevin Savage from World Vision International was engaged in a study design, conception, and early study management.

## References

[1] Pillai A Nayak A Tiwari D Pillai PK Pandita A Sakharkar S. COVID-19 disease in under-5 children: current status and strategies for prevention including vaccination. Vaccine. (2023) 11:693. doi: 10.3390/vaccines11030693, PMID: 36992278 PMC10058749

[2] Osendarp S Akuoku JK Black RE Headey D Ruel M Scott N. The COVID-19 crisis will exacerbate maternal and child undernutrition and child mortality in low-and middle-income countries. Nat Food. (2021) 2:476–84. doi: 10.1038/s43016-021-00319-4, PMID: 37117686

[3] Integrated Food Security Phase Classification. South Sudan: acute food insecurity situation January 2020 and projections for February–April 2020 and May–July 2020. (2020). Available at: https://www.ipcinfo.org/ipc-country-analysis/details-map/en/c/1152422/?iso3=SSD (Accessed April 2, 2023).

[4] Food Security Information Network, Global Network Against Food Crises. Global report on food crises, 2021 (2021). Available at: https://www.wfp.org/publications/global-report-food-crises-2021 (Accessed April 13, 2023).

[5] UN Office for the Coordination of Humanitarian Affairs. South Sudan humanitarian needs overview 2021 (2021). Available at: https://reliefweb.int/report/south-sudan/south-sudan-humanitarian-needs-overview-2021-january-2021 (Accessed April 13, 2023).

[6] UNICEF, Global Nutrition Cluster, Global Technical Assistance Mechanism for Nutrition. Management of child wasting in the context of COVID-19. Brief No. 1 (2020). Available at: https://www.nutritioncluster.net/resources/management-child-wasting-context-covid-19-brief-no1-march-27th-2020 (Accessed April 13, 2023).

[7] South Sudan Nutrition Cluster. South Sudan guidance for nutrition service delivery in context of COVID 19 (2020). Available at: https://www.nutritioncluster.net/Guidance_For_Nutrition_Service_Delivery_In_Context_Of_COVID_19 (Accessed April 13, 2023).

[8] UNICEF. Rapid review: screening of acute malnutrition by the family at community level (2020). Available at: https://www.unicef.org/documents/rapid-review-screening-acute-malnutrition-family-community-level (Accessed April 2, 2023).

[9] Blackwell N Myatt M Allafort-Duverger T Balogoun A Ibrahim A Briend A. Mothers understand and can do it (MUAC): a comparison of mothers and community health workers determining mid-upper arm circumference in 103 children aged from 6 months to 5 years. Arch Public Health. (2015) 73:26. doi: 10.1186/s13690-015-0074-z, PMID: 25992287 PMC4436117

[10] Alé FG Phelan KP Issa H Defourny I Le Duc G Harczi G. Mothers screening for malnutrition by mid-upper arm circumference is non-inferior to community health workers: results from a large-scale pragmatic trial in rural Niger. Arch Public Health. (2016) 74:38–2. doi: 10.1186/s13690-016-0149-5, PMID: 27602207 PMC5011948

[11] Bliss J Lelijveld N Briend A Kerac M Manary M McGrath M. Use of mid-upper arm circumference by novel community platforms to detect, diagnose, and treat severe acute malnutrition in children: a systematic review. Glob Health Sci Pract. (2018) 6:552–64. doi: 10.9745/GHSP-D-18-00105, PMID: 30185435 PMC6172115

[12] Rao B Roby R LeBeau K du Cros P Briend A Fritsch P. Comparing the accuracy and sensitivity of a double-sided universal MUAC strap (“uniMUAC”) with the UNICEF MUAC strap. F1000Res. (2017) 6:885. doi: 10.7490/f1000research.1114216.1

[13] Grant A Njiru J Okoth E Awino I Briend A Murage S. Comparing performance of mothers using simplified mid-upper arm circumference (MUAC) classification devices with an improved MUAC insertion tape in Isiolo County, Kenya. Arch Public Health. (2018) 76:11–9. doi: 10.1186/s13690-018-0260-x, PMID: 29484177 PMC5822476

[14] UNICEF. Product specification sheet (for local and regional procurement): child MUAC tape (2020). Available at: https://www.unicef.org/supply/media/4001/file/MUAC-tape-child-specification-May2020.pdf (Accessed April 2, 2023).

[15] GOAL. MAMI mid-upper arm circumference (MUAC) tapes (2020). Available at: https://www.ennonline.net/mamimuactapes (Accessed April 2, 2023).

[16] Isanaka S Berthé F Nackers F Tang K Hanson KE Grais RF. Feasibility of engaging caregivers in at-home surveillance of children with uncomplicated severe acute malnutrition. Matern Child Nutr. (2020) 16:e12876. doi: 10.1111/mcn.12876, PMID: 31336045 PMC7038908

[17] ReliefWeb. UNICEF introduces disposable MUAC tapes for children amid fears of COVID-19 transmission (2020). Available at: https://reliefweb.int/report/world/unicef-introduces-disposable-muac-tapes-children-amid-fears-covid-19-transmission-enar (Accessed May 28, 2023).

[18] UNICEF. Guidance note: new design for the mid-upper arm circumference (MUAC) tape (2020). Available at: https://www.ennonline.net/attachments/3644/MUAC_new_guidance_final.pdf (Accessed May 28, 2023).

[19] Rana R Barthorp H McGrath M Kerac M Myatt M. Mid-upper arm circumference tapes and measurement discrepancies: time to standardize product specifications and reporting. Glob Health Sci Pract. (2021) 9:1011–4. doi: 10.9745/GHSP-D-21-00273, PMID: 34933994 PMC8691892

[20] South Sudan SMART Surveys, 2014–2021. (2019). Available at: https://www.humanitarianresponse.info/en/operations/south-sudan/document/smart-survey-database-2018-and-2019 (Accessed May 3, 2023).

