# Supplementary material for: Caregiver use of MUAC tapes in South Sudan: a three-group prospective comparison
Source: Front Nutr. 2024 Feb 6;11:1324063. doi: 10.3389/fnut.2024.1324063 (PMC10877034; doi:10.3389/fnut.2024.1324063)
Supplement: Supplementary file 1 [file Data_Sheet_1.PDF]

Supplemental Table 1: Caregiver Use of MUAC Tapes During Monitoring Visits and Endline by Location

			N (%)[1]	**Caregiver is Comfortable Using Tape**		**Caregiver Measurement Frequency**				**Caregivers Measuring Less Than Monthly**
				Self-reported by caregiver	Study staff perception	At least Weekly	At least monthly	At least once since last visit	Not since last visit	
Monitoring 1	**Overall**		1729 (94.7%)	98.4%	90.6%	90.7%	8.2%	0.8%	0.4%	19 (1.0%)
	**By Location**	Central Equatoria	673 (89.7%)	96.4%	83.7%	78.1%	19.2%	2.0%	0.7%	16 (2.1%)
		Warrap	1056 (98.1%)	99.7%	92.3%	97.9%	1.8%	0.1%	0.2%	3 (0.3%)
		p-value		***<0.001***	***<0.001***	***<0.001***				***<0.001***
Monitoring 2	**Overall**		1802 (98.7%)	99.7%	94.7%	91.7%	6.3%	1.5%	0.5%	33 (1.8%)
	**By Location**	Central Equatoria	727 (96.9%)	99.4%	96.1%	84.4%	10.8%	3.9%	0.8%	29 (3.9%)
		Warrap	1075 (99.9%)	99.9%	93.7%	96.1%	3.5%	0.1%	0.3%	4 (0.4%)
		p-value		0.070	**0.022**	***<0.001***				***<0.001***
Monitoring 3	**Overall**		1770 (97.0%)	99.9%	97.6%	92.2%	5.6%	2.0%	0.2%	34 (1.9%)
	**By Location**	Central Equatoria	700 (93.5%)	99.7%	96.6%	82.0%	12.2%	5.3%	0.5%	34 (4.5%)
		Warrap	1070 (99.4%)	100.0%	98.3%	98.2%	1.8%	0.0%	0.0%	0 (0.0%)
		p-value		0.088	**0.018**	***<0.001***				***<0.001***
Endline	**Overall**		1825 (100%)	99.8%	98.6%	91.8%	7.4%	0.5%	0.3%	13 (0.7%)
	**By Location**	Central Equatoria	749 (100%)	99.6%	96.5%	80.1%	17.5%	1.5%	0.9%	13 (1.7%)
		Warrap	1076 (100%)	100.0%	100.0%	97.9%	2.1%	0.0%	0.0%	0 (0.0%)
		p-value		**0.036**	***<0.001***	***<0.001***				***<0.001***

[1]Percent of households with available children measured at time point (excludes households in which all children died or permanently moved away at or before time point)
