# Supplementary material for: Caregiver use of MUAC tapes in South Sudan: a three-group prospective comparison
Source: Front Nutr. 2024 Feb 6;11:1324063. doi: 10.3389/fnut.2024.1324063 (PMC10877034; doi:10.3389/fnut.2024.1324063)
Supplement: Supplementary file 2 [file Data_Sheet_2.PDF]

Supplemental Table 2: Child MUAC Measurement During Monitoring Visits and Endline (Caregiver, Study Team, and Measurement Agreement) By Location

				Caregiver Measurement				Evaluation Team Measurement				Measurement Agreement[2]			
			N (%)[1]	Mean MUAC (SD)	Green	Yellow	Red	Mean MUAC (SD)	Green	Yellow	Red	True negative	True positive	False negative	False positive
Monitoring 1	**Overall**		2254 (93.9%)	14.6 (1.3)	98.0%	1.8%	0.2%	14.5 (1.1)	97.8%	2.0%	0.2%	97.6%	1.9%	0.4%	0.2%
	**By Location**	Central Equatoria	865 (88.2%)	14.6 (1.3)	96.2%	3.4%	0.4%	14.7 (1.2)	95.6%	4.0%	0.4%	95.3%	3.5%	0.9%	0.3%
		Warrap	1389 (97.8%)	14.2 (1.1)	98.9%	0.9%	0.1%	14.5 (1.0)	99.0%	0.9%	0.1%	98.8%	0.9%	0.1%	0.1%
		p-value		0.134		***<0.001***		***<0.001***		***<0.001***			***<0.001***		
Monitoring 2	**Overall**		2358 (98.2%)	14.9 (1.2)	98.5%	1.4%	0.0%	14.7 (1.1)	98.4%	1.4%	0.2%	98.3%	1.3%	0.3%	0.2%
	**By Location**	Central Equatoria	941 (95.9%)	15.0 (1.3)	98.5%	1.5%	0.0%	14.9 (1.2)	98.4%	1.3%	0.3%	98.2%	1.3%	0.3%	0.2%
		Warrap	1417 (99.8%)	14.3 (1.0)	98.5%	1.4%	0.1%	14.5 (1.0)	98.4%	1.5%	0.1%	98.3%	1.3%	0.2%	0.1%
		p-value		**0.003**		0.709		***<0.001***		0.329			0.931		
Monitoring 3	**Overall**		2325 (96.9%)	15.0 (1.1)	98.8%	1.1%	0.1%	14.5 (1.0)	98.8%	1.1%	0.1%	98.6%	1.0%	0.2%	0.2%
	**By Location**	Central Equatoria	916 (93.5%)	15.0 (1.1)	98.4%	1.4%	0.2%	14.9 (1.1)	98.1%	1.6%	0.2%	97.9%	1.4%	0.4%	0.2%
		Warrap	1409 (99.2%)	14.3 (0.7)	99.1%	0.9%	0.0%	14.3 (0.9)	99.2%	0.8%	0.0%	99.1%	0.8%	0.0%	0.1%
		p-value		**0.002**		0.115		***<0.001***		**0.034**			**0.035**		
Endline	**Overall**		2400 (100%)	14.8 (1.1)	99.4%	0.5%	0.1%	14.5 (1.0)	99.2%	0.7%	0.1%	99.2%	0.6%	0.2%	0.0%
	**By Location**	Central Equatoria	980 (100%)	14.8 (1.1)	99.5%	0.2%	0.3%	14.9 (1.1)	99.0%	0.8%	0.2%	99.0%	0.5%	0.5%	0.0%
		Warrap	1420 (100%)	14.3 (0.8)	99.4%	0.6%	0.0%	14.3 (0.9)	99.4%	0.6%	0.0%	99.4%	0.6%	0.0%	0.0%
		p-value		**0.040**		**0.035**		***<0.001***		0.204			**0.025**		

[1]Percent of available children measured at time point (excludes children that died or permanently moved away at or before time point); [2]comparison of caregiver measurement to study team gold standard based on category, where green (>12.5cm) is not wasted and yellow (11.5-12.5cm)/red (<11.5cm) is wasted; true negative=children correctly identified as not wasted (specificity), true positive=children correctly identified as wasted (sensitivity), false negative=children incorrectly identified as not wasted, false positive=children incorrectly identified as wasted.
